# Air Pollution and Odor in Communities Near Industrial Swine Operations

**DOI:** 10.1289/ehp.11250

**Published:** 2008-06-05

**Authors:** Steve Wing, Rachel Avery Horton, Stephen W. Marshall, Kendall Thu, Mansoureh Tajik, Leah Schinasi, Susan S. Schiffman

**Affiliations:** 1 Department of Epidemiology, School of Public Health, University of North Carolina, Chapel Hill, North Carolina, USA; 2 Department of Anthropology, Northern Illinois University, DeKalb, Illinois, USA; 3 Department of Health and Sustainability, University of Massachusetts Lowell, Lowell, Massachusetts, USA; 4 Department of Psychiatry, Duke University, Durham, North Carolina, USA

**Keywords:** agriculture, air pollution, community-based participatory research, environmental justice, epidemiology, quality of life, rural health

## Abstract

**Background:**

Odors can affect health and quality of life. Industrialized animal agriculture creates odorant compounds that are components of a mixture of agents that could trigger symptoms reported by neighbors of livestock operations.

**Objective:**

We quantified swine odor episodes reported by neighbors and the relationships of these episodes with environmental measurements.

**Methods:**

Between September 2003 and September 2005, 101 nonsmoking volunteers living within 1.5 mi of industrial swine operations in 16 neighborhoods in eastern North Carolina completed twice-daily odor diaries for approximately 2 weeks. Meteorological conditions, hydrogen sulfide, and particulate matter ≤ 10 μm in aerodynamic diameter (PM_10_) were monitored in each neighborhood. We used mixed models to partition odor variance within and between people and between neighborhoods, and to quantify relationships between environmental factors and odor.

**Results:**

Participants reported 1,655 episodes of swine odor. In nine neighborhoods, odor was reported on more than half of study-days. Odor ratings were related to temperature, PM_10_, and semivolatile PM_10_ in standard but not mixed models. In mixed models, odor increased 0.15 ± 0.05 units (mean ± SE) for a 1-ppb increase in H_2_S, and 0.45 ± 0.14 units for a 10-μg/m^3^ increase in PM_10_ at wind speeds > 6.75 miles per hour. The odds of reporting a change in daily activities due to odor increased 62% for each unit increase in average odor during the prior 12 hr (*t*-value = 7.17).

**Conclusions:**

This study indicates that malodor from swine operations is commonly present in these communities and that the odors reported by neighbors are related to objective environmental measurements and interruption of activities of daily life.

There is a long history of medical interest in the health impacts of environmental malodor, from Hippocrates to William Farr, England’s first Registrar General. In recent decades, scientific consideration of the health consequences of malodors has increased in the context of residential exposures to malodors from municipal solid waste landfills; waste-water treatment; land application of treated sewage sludge; industrialized animal operations; and the production, storage, and transport of industrial chemicals (Schiffman et al. 2000). Environmental malodors may prompt reports of annoyance, worry, and physical symptoms (Shusterman 2001). The extent to which malodor is an aesthetic issue versus a threat to health is a subject of scientific investigation and litigation that has important implications for environmental regulation, public health, and environmental justice (Thu 1998).

Odorant compounds can affect human health via several mechanisms (Schiffman et al. 2000; Shusterman 1992). First, at concentrations high enough to stimulate the trigeminal nerve, odorant chemicals may produce irritation of the eyes, nose, and throat, or other toxicologic effects. In this case, the toxicologic properties of the odorous molecules, rather than odor, produce symptoms. Second, via innate aversion, conditioning, or stress responses, odorant compounds can induce symptoms such as nausea, vomiting, headaches, stress, negative mood, and a stinging sensation at concentrations higher than the olfactory nerve threshold but below the trigeminal nerve threshold (Schiffman 1998; Schiffman et al. 2000; Shusterman 1992, 2001; Shusterman et al. 1991). Third, symptoms occurring in response to odorant mixtures may be due to a nonodorant component such as endotoxin, which can induce inflammation and airflow obstruction (Kline et al. 1999).

Odors may be quantified in natural settings or by laboratory analysis of ambient air samples using trained odor panels, scentometers, olfactometers, or electronic noses (Schiffman et al. 2001, 2005); however, transient and unpredictable odors are difficult to quantify. Although spontaneous reports of malodor may be quantified (e.g., Aitken and Okun 1992; Drew et al. 2007), this approach mixes variation in odor with variation in people’s propensities to report odors and the limited availability of public agencies or researchers to track reports.

Research on malodors from concentrated animal feeding operations (CAFOs) and the consequences of these malodors for the health and quality of life of nearby neighbors has increased with expansion of industrial animal agriculture. Recent studies report that CAFO neighbors experience elevated levels of gastrointestinal and respiratory tract symptoms (Thu et al. 1997; Wing and Wolf 2000), wheezing and asthma (Merchant et al. 2005; Mirabelli et al. 2006; Radon et al. 2007), and decreased secretion of salivary IgA during episodes of high odor (Avery et al. 2004). Research on malodor is of interest in the context of broader impacts of industrial livestock production on energy use, diet, air and water pollution, and occupational health and safety (Donham et al. 2007; Thu 2002).

The purpose of this study was to quantify the reports of hog odors made by neighbors of swine CAFOs. To address a common limitation of research into connections between odor and health based on self-report without objective measures, we measured hydrogen sulfide, a product of anaerobic decomposition of hog waste, and particulate matter ≤ 10 μm in aerodynamic diameter (PM_10_)_,_ which can transport odorant chemicals (Bottcher 2001); at the same time participants rated the strength of hog odor. Swine CAFOs are located disproportionately in low-income communities of color (Wilson et al. 2002; Wing et al. 2000), where fear of reprisals and community discord may discourage residents from reporting malodors and health concerns to health or environmental officials (Wing 2002), thus limiting the possibility of obtaining data about odor from public records. The Community Health Effects of Industrial Hog Operations study used community-based participatory research methods to increase the completeness and quality of data collection while promoting community organizing for environmental justice (Wing et al. 2008).

## Materials and Methods

### Setting and data collection

From September 2003 through September 2005 we collected data in eastern North Carolina, an area with one of the world’s highest densities of swine production. Volunteers were recruited through community-based organizations. Nonsmoking adults ≥ 18 years of age who lived within 1.5 mi of at least one swine CAFO and had a freezer in their home (for storage of saliva samples) were eligible to be enrolled. Participants in each neighborhood attended a structured training session at which they practiced data-collection activities. Odor sensitivity threshold was evaluated by asking participants to choose which of two vials had an odor; one vial contained distilled water and the other contained butanol. Participants were presented up to 12 pairs of vials in series. The concentration of butanol increased 2-fold with each successive pair, beginning with 10 ppm. We defined odor sensitivity as the lowest concentration of a series of five correct choices.

Twice daily for 2 weeks (three neighborhoods chose to continue up to 7 additional days) participants sat outside their homes for 10 min at times agreed upon during the training session, usually morning and evening. They used a structured diary to report the strength of hog odor and information about health and quality of life. During their 10 min outside, participants were asked to recall the strength of hog odor inside at home, outside at home, and away from home for each hour of the day since their last diary entry. In this study we examined the ratings of hourly outdoor odor as well as hourly indoor odor reported in this portion of the diary. Participants also rated the current strength of hog odor at the end of the 10-min period. We analyzed these twice-daily odor ratings, which were made in the same locations at preselected times of day, in relation to odor sensitivity and environmental variables. Odor was rated on a 9-point scale from 0 (none) to 8 (very strong). Participants also indicated whether they had changed activities or decided not to do something because of hog odor.

We placed a small farm trailer with air monitoring equipment in each neighborhood. Locations were chosen to be as inconspicuous as possible but free from trees or structures that could affect air flow. We used a tapered element oscillating microbalance ambient particulate monitor Series 1400a with a Series 8500 filter dynamics measurement system (Rupprecht and Patashnick Co, Inc., East Greenbush, NY) to record hourly values of PM_10_ and semivolatile PM_10_. Semivolatile particles are composed of compounds that simultaneously have meaningful concentrations in both vapor and condensed phases. PM_10_ values were updated every 6 min. An MDA Scientific single point monitor (Zellweger Analytics, Inc., North America, Lincolnshire, IL) provided concentrations of H_2_S (parts per billion) averaged over 15-min intervals. Temperature, humidity, wind speed, and wind direction were recorded every 10 min with a Vantage Pro Weather Station (Davis Instruments, Hayward, CA), and every 30 min with a Young Model 05103VM-42 Wind Monitor (R.M. Young Company, Traverse City, MI). The Davis wind speed data were more complete, but the instrument was less sensitive, with values about 2 mi/hr (mph) lower than the Young monitor. To fill in missing data from each machine, values from the two machines were collectively categorized as low (≤ 0.57 mph), medium (0.58–6.75 mph), or high (> 6.75 mph). In four communities, data were missing for both weather instruments for some periods. In these cases, which comprise about three percent of total records, data were obtained from the nearest airport weather station, which was about 4.5 mi away for three communities and 18.5 mi away in one.

In each neighborhood a local “community monitor” was shown how to check the operation status of the monitoring equipment and was asked to call research staff on a toll-free line to report any outage or error message. In 12 neighborhoods a study participant served in this capacity.

We calculated the number of swine CAFOs within 2 mi of the monitoring platform using latitude and longitude coordinates derived from online satellite imagery and operating permits issued by the North Carolina Division of Water Quality (Raleigh, NC). Although we used 1.5 mi as the criterion for study elgibility, we counted operations within 2 mi because *a*) odor reports are made from that far away; *b*) that distance has been used in previous research (Thu et al. 1997; Wing and Wolf 2000); and *c*) excess wheezing symptoms have been reported as far as 3 mi from swine CAFOs (Mirabelli et al. 2006). Coordinates for the monitoring trailer and each participant’s home were determined using a hand-held global positioning system device.

Following input and approval from the Community Research Advisory Board of the Concerned Citizens of Tillery (Tillery, NC) the study protocol and survey instruments were approved by the University of North Carolina’s Institutional Review Board for research involving human subjects, which follows national and international standards. All participants gave informed consent. We obtained a Certificate of Confidentiality from the National Institutes of Health because of legal measures taken by the North Carolina Pork Council to obtain identifiable participant information from a prior study (Wing 2002).

### Statistical analysis

We evaluated relationships between environmental measurements and twice-daily odor by stratification, standard linear regression, and linear mixed models. We chose the measure of twice-daily odor for these analyses because these odor ratings were provided in real time and at preselected periods, and therefore should be less susceptible to recall bias than ratings of hourly odor since the previous diary entry. The sample sizes for these analyses varied based on the numbers of missing values for environmental measurements. Although hog-odor ratings were highly right-skewed, the number of observations was adequate to produce normal sampling distributions for the regression coefficients (Lumley et al. 2002); therefore, untransformed odor was considered as a continuous dependent variable in our linear regression models. Hourly average H_2_S, temperature, humidity, and wind speed for hours centered at the time of sitting outside were considered as predictors of odor. We considered H_2_S levels for hours when all measurements were below the detection limit of 2 ppb to be zero.

Mixed models with twice-daily odor as the dependent variable and environmental measures as independent variables were fit using the SAS MIXED procedure (SAS Institute Inc., Cary, NC) to account for variance within people, between people, and between neighborhoods. We compared Akaike information criterion (AIC) statistics for fixed-slope and random-slope models and chose models with lower AIC statistics for presentation. We fit models with intercepts when the only predictor of odor is coded as an indicator variable, providing a test of the difference between the omitted category and the other category or categories. For models with the interaction of a variable coded as continuous and one coded as an indicator, we fit models with no intercept to provide an estimate of the effect of the continuous variable, its SE, and a test of difference from zero, at each level of the indicator variable.

We used mixed logistic regression for analyses of activity limitation as the dependent variable. Average hourly outdoor odor since the previous diary entry was the independent variable. Models were fitted using the SAS GLIMMIX procedure. Random intercepts and fixed effects of average odor ratings of 1 to < 2, 2 to < 3, 3 to < 5, and ≥ 5 compared with no odor were estimated as predictors of activity limitation due to odor, coded as a 0/1 variable. A model was also fit with average hourly odor as a continuous variable.

SEs of regression coefficients are presented as measures of precision in order to reduce the probabilistic interpretations implied by the use of confidence intervals. For the same reason, we assessed contributions of predictors to the fit of models by *t*-tests instead of *p*-values because this is not a randomized study (Greenland 1990).

## Results

### Neighborhood and participant characteristics

A total of 102 volunteers from 16 neighborhoods enrolled in the study. One person who had difficulty with the study protocol was excluded from analyses. Analyses here include 84 people who collected data for 2 weeks, 15 (from three neighborhoods) who chose to continue an additional 4–7 days, and 2 who stopped before 2 weeks. Sixty-six women and 35 men participated. Age ranged from 19 to 89 years, with a mean age of 53. Eighty-four participants identified themselves as black, 15 as white, one as black/Native American, and one as Latino.

Characteristics of study neighborhoods, labeled A–P, are given in Table 1. Two neighborhoods had one swine CAFO within 2 mi of the monitoring trailer, and six neighborhoods had ≥ 10 within 2 mi. Approximately two-thirds of participants lived in neighborhoods within 2 mi of ≥ 5 swine CAFOs. In nine neighborhoods, participants reported outdoor swine odor on more than half the study days. Mean temperature on study days ranged from 47°F in neighborhood A to 82°F in neighborhood K; no neighborhoods participated during January. Mean H_2_S was 0.004 ppb in neighborhood E, where 99.8% of readings were below the detection limit (2 ppb). Neighborhoods O and C had the highest mean values, 1.02 and 1.48 ppb, respectively, and the highest values recorded in neighborhood O were at the upper limit of detection, 90 ppb. Average PM_10_ varied from 10.8 μg/m^3^ in neighborhood A to 28.7 μg/m^3^ in neighborhoods C and E, whereas semivolatile PM_10_ was highest (9.2 μg/m^3^) in neighborhood O and lowest in H (−3.2 μg/m^3^), indicating the high degree of measurement error when using the microbalance to characterize semivolatile particle levels over short time periods.

### Frequency, magnitude, and duration of odor episodes

We calculated the average daily odor that participants reported following the twice-daily preselected 10-min periods of sitting outdoors, as well as the average hourly outdoor odor reported each day. Study participants collected data on 1,495 days, although twice-daily odor was missing for 39 of these days. Results for the 1,456 days with twice-daily odor information are reported here (Table 2). The average twice-daily odor was zero for 563 days (38.7%), and > 5 on 51 days (3.5%). Average hourly outdoor odor was zero for 591 days (40.6%) and > 5 on 33 days (2.3%). Average twice-daily odor was zero on fewer days than average hourly odor. This is possible because participants could report nonzero odor during twice-daily times sitting outdoors when there was no odor at other times during the hour.

**Figure 1:**
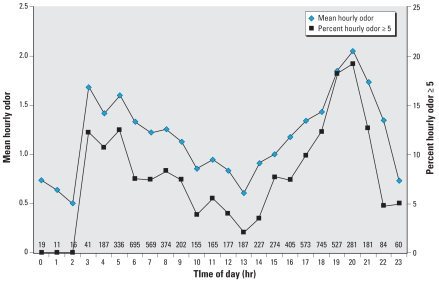
Time of day and odor. Numbers above the *x*-axis indicate the number of hourly ratings for that time point.

Reported hourly outdoor odor was highest in the mornings and evenings and lowest in the middle of the day and night (Figure 1). Morning odor was highest around 0300 hours (mean = 1.7) when 12.2% of ratings were ≥ 5. Mean hourly odor was 2.1 at 2000 hours, when 19.2% of odor ratings were five or greater.

Based on hourly outdoor odor ratings, participants reported 1,655 odor episodes (Table 3). The duration of an episode is the number of consecutive hours that swine odor was reported to be above zero. The majority of episodes (62.1%) lasted 1 hr, whereas 9 episodes (0.5%) lasted ≥ 9 hr. Average odor was < 2 for about 39% and > 5 for about 16% of odor episodes lasting 1 or 2 hr. Average strength was ≥ 5 for > 21% of odor episodes of ≥ 3 hr.

Hog odor was reported inside homes on 185 of 1,456 person-days of follow-up (12.5%). Five hundred episodes of indoor hourly odor were reported, of which 233 (46.6%) lasted 1 hr, 179 (35.8%) lasted 2–3 hr, and 88 (17.6%) lasted ≥ 4 hr. Three of the 1-hr indoor odor episodes, rated 3, 6 and 8, were reported in the middle of time periods when consistent sleep was indicated.

Butanol odor sensitivity threshold was estimated for 98 participants, of whom 39 had a threshold of 10 or 20 ppm (Table 4). Most odor ratings were provided by people with butanol detection thresholds between 10 and 160 ppm. Average reported odor declined with sensitivity from 20 to 160 ppm. Among the 12 participants with odor thresholds of ≥ 320 there was not a clear relationship between odor sensitivity and average odor.

### Environmental correlates of odor

Analyses of environmental correlates were based on the twice-daily odor ratings reported at preselected times of day when participants sat outdoors for 10 min. Table 5 provides results of bivariate simple linear regression models for each environmental variable as a predictor of 10-min odor ratings. Odor ratings increased 0.26 ± 0.02 (mean ± SE) for every 10°F increase in temperature; the *t*-test value is large (11.65). Odor ratings increased 0.17 ± 0.02 for every 1-ppb increase in H_2_S, 0.04 ± 0.02 for a 10-μg/m^3^ increment in PM_10_, 0.03 ± 0.01 per 1 μg/m^3^ of semivolatile PM_10_, and 0.06 ± 0.02 for a 10% increase in relative humidity. Average odor at moderate wind speeds was 1.02. Compared with moderate wind speeds, odor was higher by 0.43 ± 0.08 at low wind speeds and higher by 0.72 ± 0.15 at high wind speeds.

Temperature and semivolatile PM_10_ showed little association with 10-min odor ratings as main effects in mixed models (data not shown). Table 6 presents effect parameters from mixed models with other environmental variables. The relationship between H_2_S and odor was best fit with a random-intercept, random-slope model, in which odor increased 0.15 ± 0.05 (mean ± SE) for every 1-ppb increase in H_2_S (*t*-value for H_2_S = 3.10).

Because there is a strong main effect for H_2_S, we considered odor sensitivity as a modifier of its association with odor. H_2_S was positively related to odor among participants with detection thresholds of ≤ 160 ppm (0.17 ± 0.06/1 ppb, mean ± SE), but not among participants with thresholds of ≥320 ppm (0.02 ± 0.14/1 ppb).

The relationship between wind speed and odor was adequately fit with a random-intercept, fixed-slope model. Parameters for low and high wind speeds were estimated in mixed models with medium wind speed as the referent (Table 6). Average odor was lowest at medium wind speed (1.23 ± 0.20, mean ± SE). Compared with the odor at medium wind speed, odor was higher by 0.18 ± 0.07 units at low wind speeds and by 0.38 ± 0.13 units at high wind speeds.

Relationships between odor, H_2_S, and PM_10_ depended on wind speed (Table 6). A mixed model with fixed effects for wind speed and random effects for H_2_S showed that H_2_S and odor were not associated at medium wind speed (−0.09 ± 0.10/1 ppb, mean ± SE). At low wind speeds, odor increased 0.28 ± 0.11/1 ppb (*t* = 2.49), and at high wind speed there was an increase of 0.77 ± 0.44/1 ppb (*t* = 1.75). In contrast, PM_10_ was associated with odor at high wind speeds (0.45 ± 0.14/10 μg/m^3^; *t* = 3.14), but not at low or medium wind speeds.

### Activity limitation

On 118 occasions 34 participants reported that they cancelled or changed an activity because of hog odor. Typical changes included closing windows, avoiding sitting in the yard and socializing with friends, cancelling plans to barbecue, not putting clothes out to dry, declining exercise via outdoor walks, not putting up Christmas lights, not being able to garden or mow the lawn, not washing the car, or not being able to sit on the porch. One participant reported on two occasions that odor made it difficult to sleep. Whereas in other records this participant reported 6–8 hr of sleep during the previous night, on these two occasions he or she indicated having slept either 0 or 4 hr. The common theme in these disruptions was the adverse impact of odor on people’s social and personal space. There was an association between activity change and average outdoor odor intensity during the 12 hr prior to a diary record, with odor grouped into several levels (Table 7). Participants noted changes in activity due to odor from 1.4% of occasions when average odor was < 1.0 up to 16.2% when average odor was ≥ 5.0. Estimates from logistic mixed models with random intercepts and a fixed slope for odor show a similar relationship; all model coefficients are substantially larger than their SEs, and *t*-values are large. A separate model was estimated for odor as a continuous variable; the log odds ratio of activity change for a one-unit increase in odor is 0.48 ± 0.07, a 62% increase in the odds of activity change per odor unit (*t* = 7.17).

## Discussion

In the present study 101 participants from 16 neighborhoods in eastern North Carolina reported on the strength of hog odor inside and outside their homes for approximately 2 weeks while temperature, humidity, wind speed, H_2_S, and PM_10_ were monitored nearby. One to 16 swine CAFOs were located within 2 mi of the monitoring platform in each neighborhood. Odor was reported outside on more than half the study days in 9 neighborhoods. Odor ratings made during 10-min periods of sitting outside twice a day were associated with weather conditions, H_2_S, and PM_10_. One-third of participants reported ceasing or changing their activities due to malodor, and the intensity of odors reported between diary entries was strongly associated with these reports. This study indicates that malodor from swine operations is commonly present in these communities and that the odors reported by neighbors are related to objective environmental measurements.

Neighborhoods were included in the study if at least several members were interested in participating in a 2-week study that required a 3-hr training session and a twice-daily routine of reporting and measurement. Neither the neighborhoods nor participants are a representative or systematic sample of the region. We relied on local knowledge to select neighborhoods where hog odor had been reported to community organizers and where individuals might be interested in participating. However, there are > 2,000 swine CAFOs in the region, and we had no way to identify those CAFOs with higher releases of odorant chemicals. Although it is unlikely that neighborhoods with the highest exposures were included in this study, neighborhoods with no odor problems, if they exist, would not have been included either. Pollution levels and odor strength in this study may also have been affected by actions taken by operators of swine CAFOs near the study sites; participants in several neighborhoods reported cessation or relocation of hog waste sprayers, as well as reduced odor, during their period of study participation.

Other analyses indicated that the completeness and consistency of data in this study were high (Schinasi 2007). Participants reported twice-daily odor ratings in 94% of 2,949 total journal entries and at least one such rating on 97% of 1,495 study days. On the 1,456 study days with at least one twice-daily odor rating, the mean and median percentages of hours of the day for which hourly odor ratings were provided were 96% and 100%, respectively. On 95% of study days, participants reported information on whether hog odor had altered their daily activities.

We evaluated the hypothetical possibility that, due to their access to the H_2_S monitor, odor ratings of 12 study participants who were asked to check for malfunctions with the environmental monitoring equipment could have been influenced by the value on the display screen; in this case the relationship between H_2_S and odor might be over-estimated. We refit the random-intercept, random-slope model for H_2_S and odor excluding these 12 participants; the β coefficient and its SE rounded to the same values reported in Table 6.

Although the structured reporting of odor by neighbors of swine CAFOs is a strength of our study, the frequency, duration, and intensity of reported hog odor episodes must be interpreted in the context of participants’ daily activity patterns. Participants reported being indoors at home 30.0%, outdoors at home 17.1%, away from home 25.5%, and sleeping 27.4% of hours in the study. The large proportion of time spent indoors and away from home limits information on outdoor odor episodes. The duration of outdoor odor episodes is also truncated by going indoors or away from home to avoid odor; this may contribute to the shorter duration of reported outdoor hourly odor episodes (62.1% lasted 1 hr) compared with indoor hourly odor (46.6% lasted 1 hr).

With the exception of PM_10_ in higher wind conditions, temperature, PM_10_, and semivolatile PM_10_ were correlated with hog odor ratings only if the within-person, between-person, and between-neighborhood structure of the data was ignored. This might reflect the lack of seasonal variation of these variables within neighborhoods sampled for only about 2 weeks, which is a limitation of the study design. H_2_S, in contrast, was strongly related to odor in mixed models. Unlike the weather variables, H_2_S levels varied markedly within neighborhoods. In a recent chamber experiment, naïve volunteers exposed to swine CAFO air with a 24 ppb concentration of H_2_S reported an average odor of 5.29 on a 0–8 scale (Schiffman et al. 2005). The predicted odor at 24 ppb in the present study, based on the linear regression function from Table 4 [odor = 1.25 + 0.17 × H_2_S (ppb)] produces a similar value of 5.33.

In theory, a stronger relationship between odor ratings and the concentration of odorant compounds should have been observed among people with a better sense of smell. We considered butanol detection threshold as a modifier of the H_2_S effect because, unlike PM_10_, it was strongly associated with odor even without taking into account the modifying effect of wind speed. The observation that this association was restricted to people with detection thresholds < 320 ppm suggests that this simple threshold test distinguishes a subgroup of participants (87.8%) who are more responsive to H_2_S.

The microbalance produced many negative values for semivolatile PM_10_, indicating large measurement error relative to the semivolatile particle signal. This reduced the power of the study to detect associations between reported odor and semivolatile compounds in particle phase, including ammonia, an important odorant chemical emitted by swine CAFOs (Lim et al. 2003; Reynolds et al. 1997; Wilson and Serre 2007). We did not have the capacity to directly measure ammonia or other odorant compounds for this study.

The presence of air pollution from swine CAFOs in neighboring communities depends on wind direction and speed. We did not evaluate wind direction because there were at least several CAFOs in different directions near most neighborhoods in the study. Wind speed was related to odor and was also a modifier of relationships between air pollution levels and the strength of odors reported by neighbors. Although odor was highest at high wind speeds, mean H_2_S levels were lowest at high wind speeds (0.05 ppb) compared with medium (0.09 ppb) and low (0.45 ppb) wind speeds. H_2_S was strongly related to odor at low wind speeds (0.28 ± 0.11/1 ppb). Although the point estimate of the odor–H_2_S relationship at high wind speeds was very large (0.77), its SE was also large (0.44), reflecting the limited range of H_2_S values and smaller sample size at higher wind speeds.

In contrast, PM_10_ was related to odor in mixed models only during periods of higher wind speed. This observation is consistent with the greater capacity of stronger winds to transport PM, and provides evidence that organic dusts from swine CAFOs may be inhaled by CAFO neighbors during higher wind conditions. Although PM_10_ is associated with a variety of health outcomes, most studies have been conducted among populations where the composition of PM is largely affected by combustion by-products and urban dusts. Although PM from animal dander, dried feces, feed, pharmaceuticals, and endotoxin is known to affect occupational health of workers in swine confinement buildings (Donham 1990, 1993; Donham et al. 1995, 2000), its effect at lower levels and among nonworker populations is poorly understood.

Among the 98 participants who answered questions about residential history, 76 grew up on farms where they had experience with animal odors, and 82 had lived in their homes for > 5 years. Thus, adaptation and loss of sensitivity to malodors from swine operations could have occurred. On the other hand, the study protocol prompted participants to pay attention to swine odors, thus, physiologic adaptation or reduced attention to odor as a means of coping may have been offset by the odor-reporting protocol. In considering the effects of odor, it is important to note that adaptation occurs most readily when there is little variation in the concentration of odorant chemicals, whereas swine odors are transient. Like other environmental agents that act as stressors, unpredictable acute odor episodes may cause more of a stress response in susceptible persons than nonepisodic stressors.

The health significance of malodorous compounds is due, in part, to diseases related to pollutants such as PM that would occur even among persons with no sense of smell. However, malodor also should be considered in the context of scientific interest in end points that are not specific diseases. For example, biological markers of exposure to or effects of toxicants, genetic markers of susceptibility, and physiologic states associated with increased risk of disease are widely recognized as relevant to understanding and improving environmental health, even though they are not specific diseases. Similarly, environmental malodor is an important subject for inquiry, not only because it may be involved in causation of specific diseases but because of its potential to affect health, considered as not merely the absence of disease, but as a state of physical, mental, and social well-being (World Health Organization 2002). Environmental malodors may be markers of agents that can produce inflammatory, immunologic, infectious, or toxicologic responses; additionally, they may affect physical, mental, and social well-being due to their psychological and cultural meaning (Schiffman et al. 2000). Odors that are viewed as unpleasant, embarrassing, or sickening may interfere with mood, beneficial uses of property, and social activities that are central to quality of life.

We found that average odor over a 12-hr period relates strongly to changes in activities because of hog odor. Both reports of activity limitations and the three reported episodes of indoor odor that occurred during the middle of time periods of sleep suggest that odor interrupted participants’ sleep in the middle of the night. Other studies have shown that the odor of feces and urine from liquid waste management systems can negatively impact neighbors’ quality of life. Among a subsample of participants in the present study, odor was found to be related to levels of stress reported in daily diaries (Horton 2007). However, numerical relationships between hog odor and disrupted activity are insufficient to capture the full impacts of quality of life disruptions. Ethnographic interviews conducted with a subsample of study participants demonstrate that malodor, when present, limited many daily physical and social activities that have been shown to reduce stress and promote health (Tajik et al. 2008). Even when odor is not present, anticipation of the potential impact of irregular and unpredictable odor events may create stress and anxiety about daily routines and about social events that could cause embarrassment if odor occurs when relatives, friends, or out-of-town guests are present (Tajik et al. 2008).

Previous studies indicate that North Carolina swine CAFOs are located disproportionately in low-income communities of color (Edwards and Ladd 2000; Ladd and Edwards 2000; Wing et al. 2000). These communities may be more adversely affected by CAFOs because of their limited resources, higher disease rates, poor food supplies, poor housing, and unprotected sources of groundwater for drinking. Lower levels of formal schooling and less access to legal and political resources make it more difficult for such communities to bring about more protective environmental policies and enforcement. The present study adds to a growing body of literature suggesting that malodor from swine CAFOs, and the physical and chemical agents with which it is associated, have the potential to negatively impact public health, especially in communities that are already vulnerable (Donham et al. 2007).

## Figures and Tables

**Table 1:** Characteristics of neighborhoods and CAFOs within 2 mi of the monitoring platform.

Site	Swine CAFOs (no.)	Participants (no.)	Mean 10-min odor	Days with any odor outdoors (%)	Days with any odor indoors (%)	Mean temp (F)	Mean H_2_S (ppb)	H_2_S values < 2 ppb (%)	Highest H_2_S (ppb)^a^	Mean PM_10_ (μg/m^3^)	Mean semivolatile PM_10_ (μg/m^3^)
A	1	7	0.4	26	2	47	0.01	99.7	4	10.8	1.1
B	1	6	0.7	48	10	50	0.09	97.0	9	13.6	1.8
C	3	5	1.4	70	14	60	1.48	77.1	28	28.7	2.7
D	3	6	0.8	68	9	59	0.41	90.7	20	13.7	1.4
E	4	7	0.5	20	15	77	> 0.00	99.8	2	28.7	5.9
F	4	4	2.7	95	46	77	0.15	94.2	10	28.4	3.9
G	5	4	0.6	41	2	51	0.07	96.7	3	17.5	5.0
H	9	6	1.0	45	9	63	0.02	98.9	3	16.8	−3.2
I	9	9	2.9	88	23	80	0.40	90.9	20	27.0	7.5
J	9	4	1.9	63	15	79	0.40	91.2	52	21.7	3.5
K	10	8	1.3	73	12	82	0.28	93.3	21	22.8	8.6
L	12	7	0.8	43	3	71	0.05	97.6	4	23.0	4.6
M	12	10	2.1	73	11	75	0.05	98.6	27	17.1	1.6
N	15	5	0.9	49	13	59	0.01	99.5	4	27.3	4.6
O	15	5	1.8	68	26	77	1.02	91.1	90	18.7	9.2
P	16	8	1.2	66	10	59	0.08	97.3	9	19.1	6.5

temp, temperature.

a Based on 15-min average values.

**Table 2:** Daily averages of twice-daily and hourly outdoor odor ratings (scale of 0–8).

Mean odor rating	Twice-daily odor [no.(%)]	Hourly outdoor odor [no. (%)]
0	563 (38.7)	591 (40.6)
> 0 to < 2	541 (37.2)	581 (39.9)
> 2 to < 5	301 (20.7)	251 (17.2)
≥ 5	51 (3.5)	33 (2.3)
Total	1,456 (100.0)	1,456 (100.0)

**Table 3:** Duration and strength of reported outdoor odor episodes.

	Duration of hourly outdoor odor episode (hr)					
Mean odor	1 [no.(%)]	2 [no.(%)]	3 [no.(%)]	4–8 [no.(%)]	≥9 [no.(%)]	Total
1 to < 2	398 (38.8)	126 (38.5)	30 (18.9)	29 (21.8)	3 (33.3)	586 (35.4)
2 to < 5	462 (45.0)	152 (46.5)	89 (56.0)	76 (57.1)	4 (44.4)	783 (47.3)
≥ 5	167 (16.3)	49 (15.0)	40 (25.2)	28 (21.1)	2 (22.2)	286 (17.3)
Total	1,027 (100.0)	327 (100.0)	159 (100.0)	133 (100.0)	9 (100.0)	1,655 (100.0)

**Table 4:** Butanol odor sensitivity threshold and mean twice-daily odor.

Butanol (ppm)	No. of participants	No. of twice-daily odor ratings	Mean odor
10	18	503	1.51
20	21	575	1.64
40	15	405	1.32
80	14	396	1.08
160	17	479	0.85
320	4	97	1.39
640	5	125	1.25
1,280	1	20	1.55
2,560	1	27	4.89
5,120	1	28	2.07
20,480	1	28	1.00

**Table 5:** Simple linear regression coefficients for environmental predictors of odor.

	No. of records	Coefficient	SE	*t*-Value
Temperature (×10)	2,772	0.26	0.02	11.42
H_2_S (ppb)	2,701	0.17	0.02	8.73
PM_10_ (10 μg/m^3^)	2,005	0.03	0.02	1.89
Semivolatile PM_10_ (μg/m^3^)	2,005	0.03	0.01	2.90
Humidity (10%)	2,772	0.05	0.02	2.91
Low wind	1,617	0.43	0.08	5.73
Medium wind (intercept)	972	1.02	0.06	16.96
High wind	183	0.73	0.15	4.87

**Table 6:** Mixed-model coefficients for environmental predictors of odor.

	Effect	SE	*t*-Value
Wind speed^a^,^b^			
Low	0.18	0.07	2.62
Medium (intercept)	1.23	0.20	6.03
High	0.38	0.13	2.91
Relative humidity ≥50%	0.29	0.11	2.59
H_2_S (ppb)^c^	0.15	0.05	3.10
H_2_S × wind speed^d^			
Low	0.28	0.11	2.49
Medium	−0.09	0.10	−0.83
High	0.77	0.44	1.75
PM_10_ (10 μg/m^3^) ×wind speed^e^			
Low	−0.01	0.05	−0.23
Medium	0.00	0.02	0.25
High	0.45	0.14	3.14

a Random-intercept, fixed-slope model.

b Low, ≤ 0.57 mph; 0.57 < medium ≤ 6.75; high, > 6.75.

c Random intercepts, random slopes.

d Random intercept, random slope for H_2_S, random intecept, fixed slope for wind.

e Random intercept, fixed slope for wind and PM_10_.

**Table 7:** Reports of change in activities due to odor in relation to average odor during the previous 12 hr.

12-hr average	No. of changes in activity reports	Percentage of times with change in activity	Rate ratio	Log_e_ odds ratio^a^	SE	*t*-Value
Odor < 1	22	1.4	1.0	Referent	—	—
1 ≤odor < 2	23	5.1	3.6	1.32	0.38	3.46
2 ≤odor < 3	19	7.1	5.0	1.56	0.40	3.93
3 ≤odor < 5	30	11.0	7.7	2.12	0.39	5.46
Odor ≥5	24	16.2	11.3	2.78	0.43	6.39

a From mixed model with random intercepts and fixed slope for odor terms.
